# From Digital Inclusion to Digital Transformation in the Prevention of Drug-Related Deaths in Scotland: Qualitative Study

**DOI:** 10.2196/52345

**Published:** 2024-09-24

**Authors:** Hadi Daneshvar, Hannah Carver, Graeme Strachan, Jessica Greenhalgh, Catriona Matheson

**Affiliations:** 1 School of Health and Social Care Edinburgh Napier University Edinburgh United Kingdom; 2 Faculty of Social Sciences University of Stirling Stirling United Kingdom

**Keywords:** digital inclusion, digital transformation, digital health, drug-related death, digitalization, drug overdose, drug overdose death, harm reduction, mobile phone, digital divide

## Abstract

**Background:**

Globally, drug-related deaths (DRDs) are increasing, posing a significant challenge. Scotland has the highest DRD rate in Europe and one of the highest globally. The Scottish Government launched the Digital Lifelines Scotland (DLS) program to increase the provision of digital technology in harm reduction services and other support services. Digital technology responses to DRDs can include education through digital platforms, improved access to treatment and support via telehealth and mobile apps, analysis of data to identify risk factors, and the use of digital tools for naloxone distribution. However, digital technology should be integrated into a comprehensive approach that increases access to services and addresses underlying causes. Digital transformation could enhance harm reduction service and support, but challenges must be addressed for successful implementation. The DLS program aims to enhance digital inclusion and improve health outcomes for people who use or are affected by drug use to reduce the risk of DRDs.

**Objective:**

This study aims to explore the role of digital technology as an enabler and supporter in enhancing existing services and innovating new solutions, rather than being a stand-alone solution. Specifically focusing on individuals who use drugs, the research investigates the potential of digital inclusion and technology provision for preventing DRDs within the context of the DLS program.

**Methods:**

Semistructured interviews were conducted with 47 people: 21 (45%) service users, 14 (30%) service providers, and 12 (26%) program staff who were all involved in DLS. Interviews were audio recorded, transcribed, and then coded. Analysis was done in three phases: (1) thematic analysis of interview data to identify the benefits of digital technologies in this sector; (2) identification of the challenges and enablers of using digital technologies using the Technology, People, Organizations, and Macroenvironment conceptual framework; and (3) mapping digital technology provision to services offered to understand the extent of digital transformation of the field.

**Results:**

Participants identified increased connectivity, enhanced access to services, and improved well-being as key benefits. Digital devices facilitated social connections, alleviated loneliness, and fostered a sense of community. Devices enabled engagement with services and support workers, providing better access to resources. In addition, digital technology was perceived as a preventive measure to reduce harmful drug use. Lack of technical knowledge, organizational constraints, and usability challenges, including device preferences and security issues, were identified.

**Conclusions:**

The study found that digital inclusion through the provision of devices and connections has the potential to enhance support in the harm reduction sector. However, it highlighted the limitations of existing digital inclusion programs in achieving comprehensive digital transformation. To progress, there is a need for sustained engagement, cultural change, and economic considerations to overcome barriers.

## Introduction

### Background

The world is grappling with a drug-related death (DRD) epidemic, affecting many countries globally. However, specific statistics for DRDs worldwide are not readily available, although there were an estimated 600,000 deaths related to drug use worldwide in 2019 [1,2]. While data vary across countries and regions, it is evident that DRDs pose a significant challenge on a global scale. Scotland has been facing a significant issue with DRDs in recent years. In 2022, a total of 1051 DRDs were reported in Scotland [3]. The DRD rate in Scotland is several times higher than in the rest of the United Kingdom and is also the highest in Europe. High DRD rates worldwide, including in Scotland, are due to complex and multifaceted issues, including poverty, a complex drug market, the proliferation of unregulated drugs, the aging population of people who use drugs, social inequality, stigma, and a lack of adequate treatment and harm reduction services [4-7].

The Scottish Government has recognized the severity of the problem [8]. It declared DRDs a public health emergency and established a Drug Deaths Taskforce in 2019 to propose solutions [9]. The Drug Deaths Taskforce and Scottish Government recognized the potential of digital technology, to provide connection and support to people at risk of overdose. This gave rise to the Digital Lifelines Scotland (DLS) program [10] which provided funding to support the provision of devices and data to enable digital inclusion and support between 2021 and 2023.

The objective of the DLS program was to enhance digital inclusion and develop digital solutions that effectively cater to the specific needs of individuals, thereby improving the health outcomes for people who use drugs or their families and thereby reducing their risk of DRDs. Digital inclusion is defined as “the activities necessary to ensure that all individuals and communities, including the most disadvantaged, have access to and use of information and communication technologies” [11]. The DLS program specifically offered different digital solutions encompassing digital devices, assistance, education, well-being services, and innovative approaches to people at risk of drug-related harm. Following the launch of the DLS program, we evaluated the impact of digital technology availability and use on service users and service provision.

Different types of digital technology have the potential to play a significant role in addressing DRDs [12]. Daneshvar et al [13] considered that digital technology has the potential to be used to help combat the problem by providing education, raising awareness about the risks of drug use, promoting harm reduction strategies, and providing educational resources to individuals and communities. Social media campaigns, websites, mobile apps, and web-based communities can all be effective tools for disseminating information and reaching a wide audience. Second, telehealth and telemedicine can improve access to addiction treatment and mental health support, particularly in rural or underserved areas where there may be a shortage of health care providers [14-16]. Telemedicine can facilitate remote consultations, medication-assisted treatment, counseling, and follow-up care. Mobile apps also have the potential to act as support, monitoring, and self-management tools for individuals experiencing drug use problem by offering features, such as tracking substance use, setting goals, connecting with support networks, accessing educational materials, and providing immediate access to crises helplines [12]. In addition, by analyzing large datasets and using predictive modeling techniques, digital technology can help identify patterns and risk factors associated with DRDs [12,17,18]. This information can assist policy makers and health care professionals in targeting interventions and allocating resources more effectively. Finally, digital tools, such as distribution apps for naloxone (an opioid overdose reversal medication) or wearable devices, can help prevent or reverse overdoses by providing timely information and resources. These technologies can facilitate the dissemination of naloxone and provide instructions on its use during an emergency, while wearable devices can detect overdose situations [12]. Overall, by promoting harm reduction, facilitating access to support services, enhancing communication and engagement, and enabling personalized interventions, digital technology has the potential to empower service users and improve their overall well-being [12].

### This Study

While digital technology holds promise, it is not a stand-alone solution. It is rather an enabler and supporter for enhancing the existing service or innovating new services [19]. This paper examines the potential of digital inclusion and provision of technology for people who use or used drugs (called service users in this study) and identifies the barriers and enablers in the use of digital technologies for preventing DRDs, as part of the larger study of the evaluation of the DLS program. To do this, we address the following research questions: (1) What are the perceived benefits of the provision of digital technology for service users and what type of services (existing or new) are offered because of the provision of digital technologies? and (2) What are the barriers and enablers for using digital technologies for prevention of DRDs?

We have adopted a qualitative research approach to examine the views of service users and providers and those delivering the DLS program.

## Methods

### Overview

This paper draws on data from a wider study that evaluated the DLS program using semistructured interviews and surveys [20]. The study was informed by the Technology, People, Organization, and Macroenvironment (TPOM) framework [21]. This framework underscores the interconnectedness between various technological and social dimensions, illustrating how diverse viewpoints influence the implementation and adoption of emerging technologies. Qualitative data relating to technology were considered.

### Participants and Recruitment

This study used purposive sampling [22] to recruit participants from 3 groups: (1) service users who had access to digital solutions via the DLS program, (2) service providers providing digital solutions through DLS funding, and (3) members of the DLS program team or board responsible for the delivery of the program. We collected data from 3 groups of participants to provide insight into the DLS program from a range of viewpoints rather than covering a single perspective.

The inclusion criteria of participants were as follows: first, service users who were using illicit drugs (or had done so in the last 12 months) who had wider health and social challenges (multiple and complex needs) and received a digital technology–based innovation funded by the DLS program. Second, the criteria included service providers who were managers, frontline staff, and volunteers working in third-sector organizations that provided digital solutions funded by the DLS program, including harm reduction services, counseling, and housing support. In the context of the United Kingdom, third-sector organizations operate distinctively from both the public and private sectors. This classification encompasses a variety of voluntary and community groups, including registered charities, as well as other forms, such as associations, self-help groups, and community-based organizations. In addition, it includes social enterprises, mutuals, and cooperatives [23]. Third, the criteria included the program delivery team and the program board, responsible for the delivery of DLS. Through purposive sampling, we ensured that participants were recruited from a range of organizations and initiatives funded by DLS (eg, types of technology and training and service delivery) with different genders, roles, and diverse types of technology received. The exclusion criteria were people aged <18 years, unable to provide informed consent, unable to speak or understand English, unable to take part due to severe mental health or behavioral problems, those not currently living in Scotland, and those not involved in DLS. We used the DLS program to identify participants in the study.

Service users were recruited via service providers, who were emailed and provided with information about the study by the DLS program team and asked to pass this on to relevant individuals. Staff in the organizations then arranged interviews with the study researchers at a suitable date or time. Service providers were recruited via email from the DLS program team, who provided them with information about the study and asked them to contact the research team to arrange an interview. The program team or program board was emailed by the research team and invited to participate, with interviews being arranged directly with the researchers.

### Semistructured Interviews

The interview guide was developed by HD, CM, and HC (Multimedia Appendix 1), incorporating relevant areas of the TPOM and drawing on insights from the broader DLS program delivery work and a prior user need study [20]. The topic guide for service user participants primarily addressed the social or human (People) domains, examining how the technology was used and its impact on service users and their relationships with service providers.

Participants were provided with an information sheet and had the opportunity to ask questions. Written or verbal informed consent was provided before each interview. All interviews were conducted between August 2022 and February 2023. Interviews took place either in person, on the web, or over phone by one of the researchers (HD, GS, and JG) and lasted an average of 32 minutes. All interviews were audio recorded. Interviews with service users explored their experiences of receiving support as part of DLS; service provider interviews covered the infrastructure of digital services and current availability and levels of digital literacy. Interviews with the program team explored their views of the impact of the program and gaps, barriers, and enablers at an organizational and macroenvironment level. Participants were provided with a debrief sheet that outlined information about the study and sources of support.

### Data Analysis

The TPOM provided a broad conceptual framework for analysis [21]. This framework is designed to cover areas of evaluation that allows for provision of both formative and summative evaluation for studying technology in health and social care settings [21]. The interviews were transcribed verbatim by an external transcriber and identifiable information was removed, with the Scottish dialect retained. Data analysis was completed using NVivo (version 12; Lumivero), which streamlines qualitative research by allowing researchers to organize, manage, and code data efficiently, including text, thereby saving time, and enhancing accuracy [24]. NVivo was chosen as it is the software regularly used by members of the research team. GS coded 3 transcripts to develop the initial coding framework, which was reviewed by HC. Because no structural changes were necessary, GS and HD proceeded to code the remaining transcripts.

Data analysis had 3 phases. In phase 1, to understand the promises of digital technologies and their transformative outcomes, we used thematic analysis [25] within the technology domain of the TPOM to identify categories of perceived benefits. The coding started in a line-by-line manner on data, and it involved understanding the “meanings” of the messages, rather than the words used to communicate [26]. We then searched for common subthemes across codes in an iterative process where we moved back and forth between the codes to identify commonalities.

**Figure 1:**
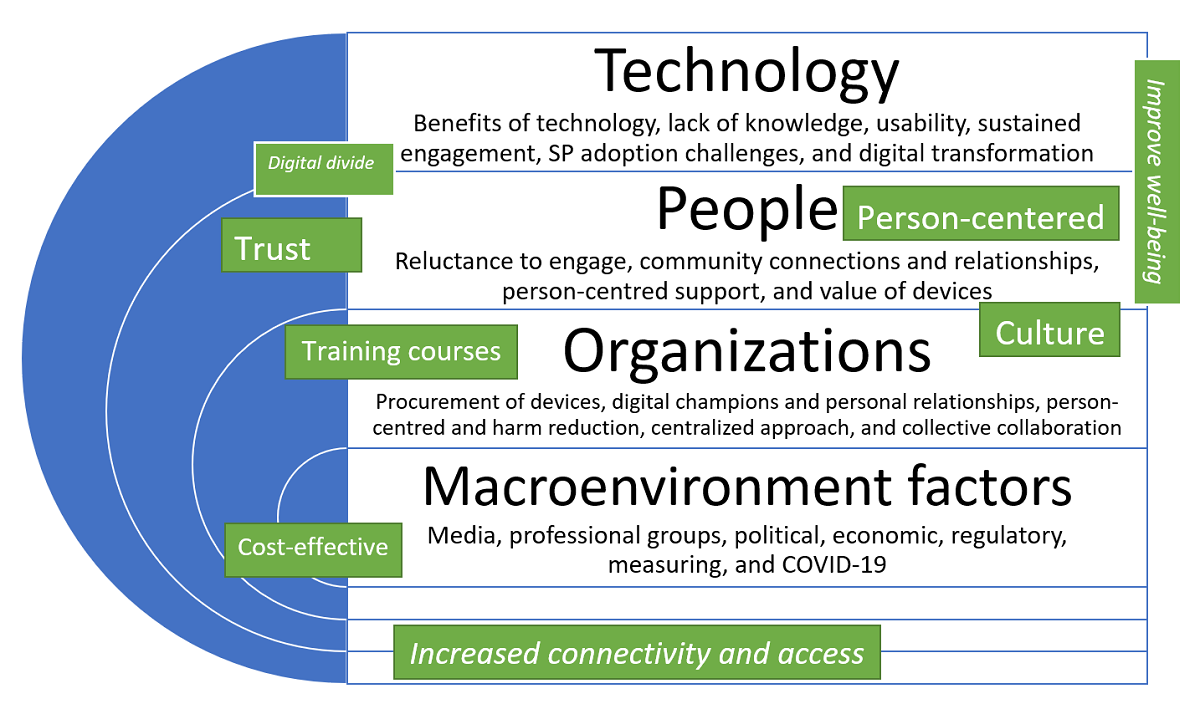
Adapted Technology, People, Organization, and Macroenvironment (TPOM) framework diagram.

In phase 2, we coded the data related to the challenges and enablers of using digital technologies for the provision of harm reduction and services. Then, in an iterative process, we grouped the codes into subthemes around challenges. In this phase, we identified the enablers in response to challenges as shown in Figure 1, where the green boxes are added to the original TPOM framework.

**Figure 2:**
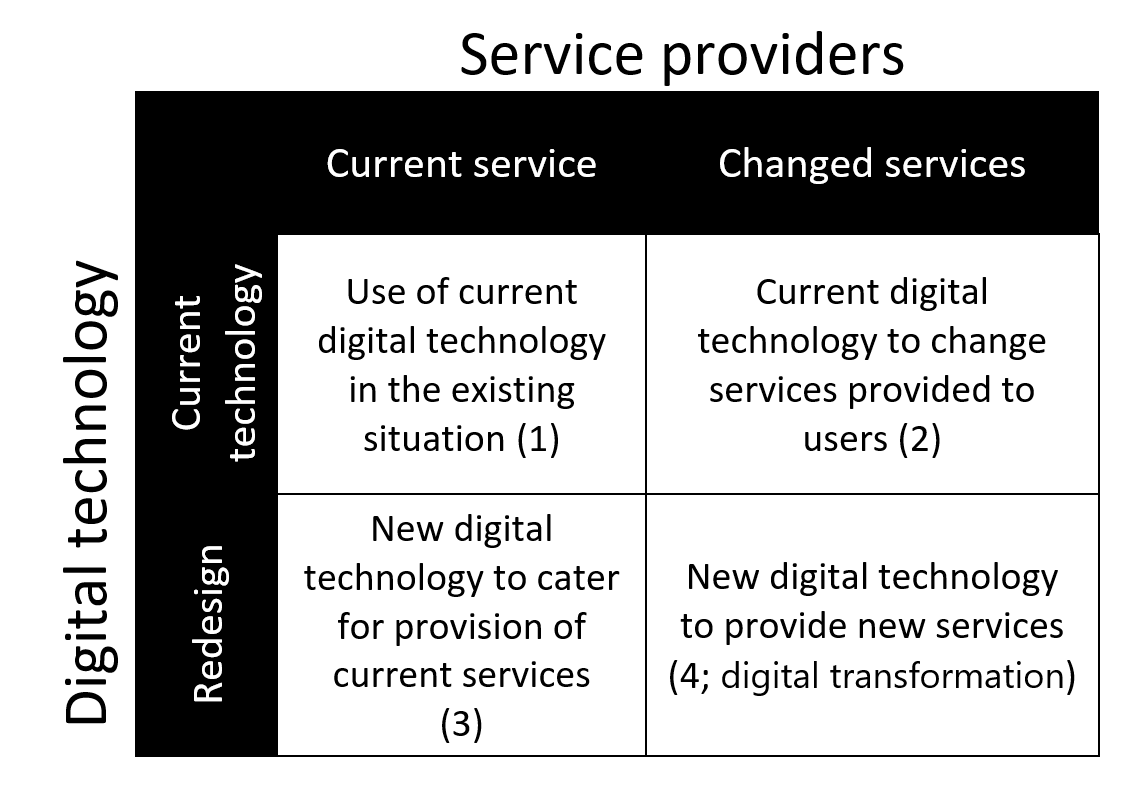
Digital technology and service providers.

In phase 3, we reviewed findings against a conceptual framework (Figure 2) by Daneshvar et al [27] to explore the relationship between existing and new digital technology and the provision of existing and new services. We used this model to categorize the potential of different types of technologies. This model encompasses 2 main components: the integration of digital tools into provision of existing services, and the creation of novel services through adoption and use of established or emerging digital tools.

### Ethical Considerations

This study adhered to the guidelines of the Declaration of Helsinki and received approval from the University of Stirling’s General University Ethics Panel (7799—August 2022), the Ethics Subgroup of the Research Coordinating Council of the Salvation Army, Turning Point Scotland, and Shine Mentoring. All participants provided informed consent either through a consent form or verbally. All data and quotes, including secondary data, were pseudonymized and attributed to each participant; identifiable names, places, or people were removed.

Service user participants received a £10 (US $13) shopping voucher as an honorarium for their participation. All data protection and General Data Protection Regulation aspects of the project and data privacy have been reviewed and approved by the Data Protection Office of Stirling University, and a Data Protection Impact Assessment form has been issued.

## Results

### Overview

The DLS program provided diverse digital solutions, including devices, support, education, and innovative services, to individuals at risk of drug-related harm. A total of 534 devices were distributed by 13 (81%) of the 16 organizations involved in the DLS program: 265 (49.6%) smartphones, 145 (27.2%) data packs, 75 (14%) tablets, and 49 (9.2%) laptops.

In total, 47 interviews were conducted by researchers, with 21 (26%) service users, 14 (30%) service providers, and 12 (45%) program team or board members. Table 1 shows the breakdown of the number of service users, service providers, and program team or board participants. The differences in the total number of participants from each organization are due to the different sizes of organizations. The demographics of participants were not collected, but they were similar to those in our related papers [20,28].

**Table 1:** Participation numbers and organizations.

Breakdown of interview participants (n=47) by organization, program team, or board member		Participation, n (%)
**Program team or board members (n=12, 26%)**		
	Program organization 1	4 (9)
	Program organization 2	2 (4)
	Program organization 3	2 (4)
	Program organization 4	2 (4)
	Program organization 5	1 (2)
	Program organization 6	1 (2)
**Service provider (n=14, 30%)**		
	Service provider 1	5 (11)
	Service provider 2	2 (4)
	Service provider 3	2 (4)
	Service provider 4	1 (2)
	Service provider 5	1 (2)
	Service provider 6	1 (2)
	Service provider 7	1 (2)
	Service provider 8	1 (2)
**Service user (n=21, 45%)**		
	Service provider 1	9 (19)
	Service provider 5	8 (17)
	Service provider 2	3 (6)
	Service provider 4	1 (2)

The findings are presented as 3 themes and 7 subthemes as presented in Textbox 1. Then, they are brought together to represent the journey of digital transformation in the sector.

**Themes and subthemes of the findings.**

**Themes and subthemes**

- The perceived and actual benefits of digital technology for service users
  - Increased connectivity for informal support
  - Increased access to service
  - Improved well-being
- Challenges of and enablers for using digital technology
  - Technological
  - People
  - Organizational
  - Macroenvironment
- The journey to digital transformation

### The Perceived and Actual Benefits of Digital Technology for Service Users

The benefits of technology provision described by participants were (1) increased connectivity for informal support, (2) increased access to services, and (3) improved well-being.

#### Increased Connectivity for Informal Support

The first category of benefits of digital devices as identified by participants was making connections with others to receive some type of support. This support could range from overcoming loneliness to creating friendships and a sense of community building:

Before I got the iPad...I wasn’t really connecting with people...But it [iPad] opened up a lot of doors for me, aye [yes]...it did. It made me feel more connected with people...I think more and more people are getting there...I know people in the community are getting connected.

Service user participant 1

This benefit was discussed most frequently by both service users and service providers, with many providing examples of connectivity to family and friends:

I stay on my own just now, my oldest son he’s in the Army, he’s 27 so I can Zoom him. My grandson, I can Zoom him...I can Zoom my other son, he’s in prison. He’s done it all the time. He’s 25...in prison, they’ve got these [computers] in the visit room. They started giving virtual visits during COVID, so we got a visit with him with Zoom.

Service user participant 21

We are asking people what they want, and mostly people want access to community. They want to be able to get on to social networks. They want to be able to interact with friends and family digitally, because their friends and family might have left [city/town], or they might have moved here from somewhere else, and they don’t have that community.

Service provider participant 5

Increased connectivity was also mentioned to be of high importance for those who had recently completed drug treatment recovery programs or those who were released from prison who were at risk of overdose if they started using drugs again:

The first kind of most important thing that they use it for is that initial contact, and that’s then how we work with them and do the project really. So, I think without a phone, and without that kind of line of contact, it’s pretty difficult. You know, I’ve had a few women that have come out (of prison) and not had contact numbers. And quite a lot of the time it means, you know, disengagement and you find them further along down the line but it’s really important initially.

Service provider participant 14

Several participants noted that if it were not for the provision of technology, many would lose any benefit obtained from having a digital connection:

See, until I get a new smartphone, I am not getting any contact wi’ [with] ma [my] house, my Mum hasnae [hasn’t] been. She had my phone number, so she can see me.

Service user participant 13

#### Increased Access to Services

The use of digital devices improved access to existing services. This was perceived as a benefit to both service users and service providers:

I do suspect as well that, again, if we’re giving people decent enough phones, they’re going to need to use them for other aspects of their life anyway and they’ll actually get quite used to them, you know. So, whether it’s speaking to your daughter, or speaking to your dealer, the phone’s quite important to you. But if at the same time, we can utilize that to be able to access those services when they’re ready and able to do so, then that for me is a win-win scenario.

Program team participant 6

An increase in access to services was likely due to several factors. First, people who were originally excluded due to lack of access to technology had an increased chance of being engaged as technology was provided to them:

We can cover things that are happening here. So, that access to those devices was quite helpful for engaging with people who normally wouldn’t get a chance to engage.

Service provider participant 4

As technology provision increased, access to support was made available to individuals, and therefore service users were better engaged with support workers:

Eighty-five percent of people in the project said that they engaged more with their support worker since having digital [devices]. And the reason for that is that the person is available to be contacted at any time and if they are in a moment of need, you know, we’re available to them and they’re available to us so it’s just that easy way of staying in contact. And that’s massive because not only is it like obviously that extra connection between the service and the person, it also means that when there is a moment of need, we’re able to help.

Service provider participant 12

Another reason that access to services was increased was that new offerings such as upcoming events could be more easily identified by service users:

[I find out about things] on Facebook and that, it keeps me connected digitally and also, I know where I can go and meet people, like events are advertised and what not.

Service user participant 1

One participant highlighted the connections between DLS and several wider policy programs of social inclusion. They highlighted that such digital inclusion programs give people more choices regarding access to resources and information to support their own self-management and preventive care:

The benefit, I guess, is to improve access and ideally outcomes for people in being able to connect in with services and support...I think it should bring in more choice to people about when and how they want to access services. So, rather than being told, ‘well, this is where you have to be at a particular time,’ you’ve more choice. I think it also should empower people more in terms of self-management and early intervention. So, rather than wait for somebody to tell you what’s wrong, you’re able to get data and information in real time that’s giving you some information around choices or decisions as you can make, and I suppose bring things into more real time.

Program team participant 10

Program team members and service providers discussed benefits from several perspectives, contrasting short- and longer-term benefits and objective versus more subjective aspects. One noted that participation in the project had led to directly intervening in several potentially fatal overdoses. Hence, delivery of prevention services was enabled through the use of technology:

We have intervened in six scenarios where there was a kind of near-death experience and we’ve managed to turn that around. That, for me, would be a great story to tell but whether we’re going to be able to tell it or not, I don’t know, we’re obviously still recruiting [participants for study].

Program team participant 6

The same participant discussed “softer,” but still “life-changing,” benefits that included service users being able to connect with services and other supports at key points in their recovery or rehousing journey:

So, you know, we’ve got a number of impacts that we’ve already [seen?], some of them are very kind of softer impacts though, you know, the stuff that’s coming out of the digital inclusion piece that [service providers] have been leading on? You know, it’s even just things like, you know, speaking to my housing officer. You know, connecting into services that were very difficult for you to connect into before. Or settle in quicker when you’re discharged from prison and maybe not linking up with your previous networks. You know, so some of those kind of softer life changing stories I think are equally valuable from a policy perspective.

Program team participant 6

Participants suggested that while not everyone who receives devices will be at risk of overdose, these devices could still have a positive impact by preventing individuals from engaging in harmful behaviors associated with drug use in the long term:

I think it’s okay that as well like a lot of people who get devices will not be someone that may have overdosed, but the device may be the thing that has stopped them taking that longer term pathway...We just need to kind of cut into like why are we doing this? Who’s it for? What are the projected long-term benefits? And equally I think being really bold about the fact that some of this stuff is a total unknown.

Program team participant 1

#### Improved Well-Being

Digital technologies are seen by participants to have significant potential in improving the well-being of the community. While the original intention of the provision of technology was the prevention of DRDs, one additional potential that was observed was improving people’s lives, which could result in reduced drug use and related harms:

I also think, to state the obvious, it shouldn’t just look at preventing drug death. It should look at supporting people’s wellbeing and ways into the community from drug use.

Service provider participant 5

For instance, when individuals were given access to technology, some tried to use it to make positive life changes, such as using the technology to learn new skills to find employment:

I think it’s just opening doors for people...for example, one guy, he came along and he’s really trying to get his life on track. He’s come along to the computer group on a Tuesday and he’s just really wanting to change, really wanting to do something. He’s in a bit of better place, he’s more stable. He came along and he’s wanting to look for jobs so that’s his benefit.

Service provider participant 9

The well-being of individuals was also perceived as improving, by enhancing their confidence through the use of technology. This in turn could lead to other benefits, such as connecting to a support network that was previously unavailable to them:

So, everyone’s really receptive of why we’re needing skills with computers, tablets, mobiles. And it’s just building their confidence as well. And it gives them that option to have that support network that they wouldn’t really have before.

Service provider participant 2

Another benefit identified in terms of well-being was that access to high-quality technology and the provision of web-based services could reduce people’s feelings of stress because they could access support more quickly than they might have previously:

So, when you compare that to only having a two-hour session, you’re spending 15, 20 minutes booting [up the equipment]. I think having good equipment and having an IT suite that actually works as it should enables people to come in and quickly do the things they want to do. That has made a massive difference. Because, anecdotally, prior to that, we had equipment that people would wait ages for things to happen, which would create a stressful environment, which would add stress on to individuals that don’t need stress adding on.

Service provider participant 2

One program team member noted the importance of and challenges in measuring the softer impacts. They also reflected that this project was relatively novel and that measures of success should be considered emergent:

...We just need to kind of cut into like why are we doing this? Who’s it for? What are the projected long-term benefits? And equally I think being really bold about the fact that some of this stuff is a total unknown. So, it’s like you can’t just give devices to like, you know, 25 people who are supported by the [service provider] and saying that is going to cut drug deaths by ten percent. There might be a point where we have some evidence base that really succinctly shows that, but it’s very, very difficult...

Program team participant 1

### Challenges of and Enablers for Using Digital Technologies

#### Overview

While the program intended to offer a range of benefits to service users by provision of technologies, not all perceived benefits were realized. This was due to numerous barriers at the individual (eg, lack of technical knowledge), organizational (eg, lack of resources), and macroenvironmental (eg, negative media impact) levels. Some of these barriers led to slow adoption of existing technologies for the services, while others led to limited innovations, which restricted new service provision. The subthemes are presented under technological, people, and organizational challenges.

#### Technological Challenges

Although benefits associated with technology provision were realized, the usability of the devices, digital technologies, and apps raised concerns. There were different preferences and feelings expressed toward different devices offered by the program. Some devices were perceived to be easier to use than others:

If somebody offered me a tablet, I’d definitely take it, because it would help me with my college work. And it would be easier, probably, to use a tablet than the Chromebook.

Service user participant 21

There were different factors affecting the usability of devices, such as age, level of education, and preexisting conditions. For instance, one participant explained how their dyslexia made it more challenging to work with laptops than tablets:

I guess tablets are just the same as the phones. I mean it’s still kind of like touchscreen. But again, with my dyslexia I’d have to get someone to set it up because my son-in-law had to set this up ‘cos I’ve not got a clue how to set phones up. But I would prefer the tablets to like the laptops as well...Because I find they are a lot easier for me to like use because there’s no buttons. And I get confused on what buttons I am pressing.

Service user participant 14

Another point that was highlighted was how “digitally savvy” an individual was. Smartphones and tablets were identified as more learnable and usable to those who were less proficient, as opposed to laptops and Chromebooks (Google LLC):

So, we did a trial last year where we did basically the same program but with 100 people. That told us that the two most popular devices for the type of people we’re supporting would be a smartphone or a tablet. Very few people would need or have use for a Chromebook really, or a laptop. The people we’re supporting tend to be at the start of their digital journey.

Service provider participant 10

Apart from devices, the operating system on the devices seemed to have an impact on the usability of technology:

I tried to [offer] a bit of mix of phones and tablets, because the operating software on the tablet, which tends to be an iPad, is quite user-friendly.

Service provider participant 4

Moreover, the need to use passwords and other security features was seen to negatively impact the usability of the technologies provided:

Sometimes logging into things can be a bit hard when you forget your passcode and that.

Service user participant 18

#### People Challenges

While many benefits were envisaged and realized by our study participants, the lack of technical knowledge among service users was the most evident challenge highlighted. This could lead to low engagement or short intervals of engagement with technology:

There are people that engage for a little while. There are people that come and say they don’t know how to work this. They can barely get online. They don’t even have an email address. They want to know how to log in to Zoom, all those kinds of stuff.

Service provider participant 4

There are also other service users who are more confident but have limited technology skills. They reported being more engaged in trying to find ways to gain the technical knowledge needed:

I’m confident to use [digital technology] but there’s certain parts like sending emails I’m not good at, I can receive emails but not send them.

Service user participant 21

To overcome the lack of knowledge, some service providers offered different types of training. They ranged from setting up email addresses to using social media:

It’s giving them the chance to set up things like email accounts, social media accounts as well, so they can keep in touch with friends and family and stuff like that. So, it is having a massive effect.

Service provider participant 2

Training could boost the confidence of service users, which could further improve their engagement with technology:

It’s just that initial fear that, “oh, I can’t do this.” But then when you show them, it’s like, “if you press that, that and that your whole world lights up.” And just by doing that you see the reaction on their faces. Like, “oh, this isn’t quite as hard as I expected it to be.”

Service provider participant 2

Some service users suggested that it would be beneficial for free training to be widely available for people to further develop their skills in using digital technology:

[They should] advertise free courses to show people how to use digital devices. Like the Job Centre, you can go in and use their computers and all that now, right? But see this scheme, where I got the tablet and stuff...

Service user participant 15

Technology provision without appropriate training could lead to a lack of engagement or even individuals selling their devices:

But the other thing with that is that we’ve found that if you just give people a smartphone and they don’t know how to use it and there’s no support and there’s no connectivity, of course, they’re going to sell it. It’s useless, it’s a lump of metal in their pocket and they can get £100 for it. If you teach somebody the value of digital [technology], and people start to realize that what digital is bringing to their lives in terms of the social connection, the benefits, the finances, the health, all of it, that is worth so much more to them than the second-hand resale price of what they would get going to a Cash Converters [pawn shop] and getting £40 for a second-hand phone.

Service provider participant 12

We identified that one of the important and challenging issues in technology provision and adoption programs is the sustainability of engagement after initial technology provision by the program:

And so, we’re now looking at, well, actually what’s the next iteration of Connecting Scotland? And if we were to do something similar, hand out kit connectivity, what else do we need to put in place around it? Now, unfortunately we don’t have the funding that we did because it was Covid funding, and lots of it has dried up. But actually, we’ve still got all that research and evidence to say, well, there’s other things you need to do around it to help support, and it’s more than just that kind of kit connectivity and then skills and support...that support word goes a lot further than I think we envisaged previously.

Program team participant 11

“Connecting Scotland” is a program aimed at supporting every citizen in Scotland to get on the web, thereby bridging the digital divide. It was launched in response to the COVID-19 pandemic, recognizing the importance of internet access in keeping people connected to their friends, family, and essential services, as well as enabling learning, work, and access to public services.

This means avoiding the disconnection of service users from the digital community and to keep them connected for the long term to ensure improvements in well-being and other related areas of their lives could be sustained:

...we should be looking for something a bit longer-term that’s right for them...And that might not be a continuation of a SIM card, that might be home broadband using social tariffs or something like that. It’s difficult because everyone is different, but what we’re really conscious of is not allowing people after the 12 months to fall off a cliff and come back round and start from square one again, because that would just be a year wasted in a way.

Service provider participant 10

This sustained engagement can lead to increased inclusion in the community and reduced DRDs. Technology was seen as part of a larger suite of options to keep people included in society:

I think the more options that we give people, the better. I think, you know, we say this all the time, but digital [technology] isn’t the way to solve drug deaths, but it’s one small part of the picture... Like the more that we can connect people, the better.

Service provider participant 7

Sustainability of engagement, however, requires an understanding of the need for cultural changes in the sector. One participant gave an example of how improved understanding of technology use can help sustainability:

It’s that ability to think critically and that ability to really reflect that we need to be giving people or helping people to understand. Now, not everybody will feel comfortable with it, so it’s finding ways into it because if you can do that, actually their ability to then use digital tools becomes much greater because they’re much more consciously aware of what they’re doing. And ultimately, then, what you would do is you would have an impact on culture and society.

Program team participant 11

Another challenge highlighted was the economic challenges faced by individuals, leading to unintended consequences, such as the need for food, leading to the potential selling of devices:

A sort of £80 resale probably isn’t really worth it, whereas a £200 resell is worth it. You know, if you’ve got nothing to eat and you’ve got no electricity, and I think there’s a real challenge that we face probably within Digital Lifelines and the broader digital connection work around the cost-of-living crisis...I had that a couple of weeks ago with a woman who came into our access hub on the Thursday and she wasn’t getting paid until the Tuesday and she didn’t want to sell her device, but the reality was she had absolutely no money. She had no money. No food. So, we were able to get her like an emergency food package to keep her going over the weekend and I said this at the last Digital Lifelines meetings, like we almost need like a personal budget to go alongside the devices. So that if people are at absolute crisis point that organizations have a little bit of like wiggle room where they can do say, let’s buy you a weeks’ worth of shopping...in order to keep hold of your device.

Service provider participant 3

However, the program team highlighted that while the selling of devices was identified as a challenge, only 10% of service users engaged in DLS either lost, sold, or had their devices stolen. Of those who did sell their devices, most sold them at pawn shops and later bought them back.

#### Organizational Challenges

A range of challenges related to the organizational adoption of technologies by service providers require careful consideration. As digital innovation is occurring fast, it is the adoption into practice that is perceived as a considerable challenge:

So, you bring out the technology, you develop apps or digital services that can do things. And the industry’s absolutely fantastic, they can develop anything. The challenge is actually to integrate it into our service delivery models and that means working with people, changing their roles, and the way they go about doing what they do, and that is very, you know, again, the human being factor is the bit that always is the most challenging but also the most rewarding.

Program team participant 6

One reason for these adoption issues is that service providers are already under pressure, lacking time and resources to spend on planning for the effective adoption of technologies:

Another of the challenges is just how much work there is going on in the field of drug death prevention at the moment and lots of frontline organizations feel completely overwhelmed...we’re a small fish in part of the whole of that pool. So, actually organizations have had the absolute best of intentions, but they’ve been pulled off from focusing on the digital inclusion element from other aspects.

Program team participant 6

This further leads to a lack of provision of digital services. While digital devices are made available to the service users, services for those at risk of drug-related harm are not effectively offered on the web:

I mean probably the best example is like we’re giving people devices, but very few of these organizations have what you would really call a true digital service...generally the public want access to an addiction service or a homelessness service and can go on and find it and I can self-refer. I can speak to someone right now when I’m in that service, I know what you’re doing with my data I know where I’m at in the pathway.

Program team participant 1

Moreover, there existed a notable gap in knowledge of service providers relating to data security concerns in the use of technologies within their organizations. For instance, the use of Google services posed potential risks to the security of service user data, and there was a lack of governance adherence to the General Data Protection Regulation.

### The Journey to the Digital Transformation of the Sector

Participants across all groups described various uses and benefits of different types of technologies provided by the program. The technologies provided included different types of devices, such as smartphones, tablets, and laptops, and internet connectivity tools, with service users generally preferring smartphones:

In terms of the device, it’s usually either a smartphone or a tablet. We have given out some Chromebooks...But I know the majority is mobile phones, probably about 60% and then 40% tablets.

Service provider participant 12

None of the participants described any type of new technology provided; that is, the technologies provided were laptops, smartphones, internet connections, and tablets; for example, new data-driven innovative apps were not provided. In addition, despite the provision of digital technologies, most benefits were described around existing services, for instance, making the existing knowledge more readily available, connecting more easily to their support workers, or being informed about different existing events. Only in cases of service users identifying new unintended use, such as learning new skills or identifying new support groups, resulted in some extent of innovative service delivery observed. Overall, by analyzing our findings against the model in Figure 2, we find that in the context of preventing DRD, the digital inclusion program offered current technologies mainly serving existing services, and only occasionally leading to changes in services.

Overall, we observed that the digital inclusion program used current technologies to enhance the provision of existing services. Interestingly, some participants highlighted that such programs were effective in their emphasis on teaching and promoting fundamental digital skills. While impactful, it does not cater for the transformation of existing services and practices, hence it is not a program of digital transformation:

I think because the Digital Lifelines work, you know, it’s about basic digital skills. It’s not necessarily about, you know, a big digital transformation or developing digital services. A lot of it is quite simple, on the surface.

Program team participant 1

Digital transformation requires rethinking and redesigning of processes, not adding a digital layer to existing cumbersome processes [29], and as the example mentioned subsequently indicates, this change of processes was not evident in many settings, neither drug-related nor other areas of health care provision:

Last time I needed to make a phone call to the doctor to try and get an appointment for my daughter, I made 135, I think, repeat phone calls...which is bonkers. And actually, what happened in that process was the receptionist took my details and said doctor will phone you back. Doctor then phoned me back within a couple of hours...But actually, what we’ve done there is instead of really thinking about a smart digital approach, is we’ve actually layered up two telephone calls and a face-to-face and increased costs rather than decreased costs...So, let’s think about that in a different way. Quite a lot of adults are now used to doing some form of digital or telehealth care.

Program team participant 11

By pointing to other areas of health and care provision and highlighting examples of transformations, participants pointed to the need for similar actions in the field of prevention of DRD. In this process, COVID-19 was seen as an accelerator of transformation:

Yes, I think because of COVID, things pushed through a lot quicker than they maybe have before because it was needs must, and you couldn’t see your GP. So, things like digital conferencing for appointments, and stuff like that, really ramped up throughout that as well. I think people through COVID have had to become acquainted with technology in a lot of ways...So, it’s how do we keep plodding ahead where appropriate and utilizing the momentum?

Program team participant 12

Some participants were aware of the need for transformative changes to service provision, while others were mainly concerned about improving the day-to-day activities using digital technologies:

You’d go into the travel agents, and you’d sit down, and you’d look through the brochures...‘this is the holiday that I really fancy’ and then you’d sit down with somebody. Actually, doing all of that online has changed it dramatically and you need to feel confident about doing that. And the supports and the people that you could have talked to aren’t always there when you need them to be there. So, that’s a complete transformation of how the travel industry has shifted its way of working. So, if we’re trying to do a transformation of how our health and care system works then we’re going to go through similar changes when we’re introducing digital into that. So, it’s not just a matter of me and you talking over a video instead of sitting down and, you know, having a coffee in a café, and blethering about it. This is a really simple digital interaction because we’re doing what we always do, we’re just using a different channel to do it. When you’re actually looking at digital services, they can completely change the way that you access something and how you actually use it, and we’ve not even scratched the surface of that within Digital Lifelines at this stage.

Program team participant 6

Hence, digital transformation needs new value creation, creating new digital services that did not exist previously in models of health and care. Such services have started to increase more recently as digital technologies are made available to those, for instance, in rural areas:

Those areas like, for example, the people in recovery, the people in the [rural area] that I was talking to you about. We weren’t able to help them in a digital way whereas we can now because we’ve got the offer of the devices and people can take them away and we can help them in that way. So, that’s a massive difference.

Service provider participant 9

As participants highlighted, digital transformation does not have to be about radical changes. Instead, even a focus on a particular niche as part of that bigger picture of transformation can improve the lives of people:

So, as somebody’s kind of working through that element of it are there particular parts of that, that by using digital services it makes it better and it makes it easier? Do you know whether it’s chat rooms or do, you know, what kinds of things it is that might actually help people at those particular little glitches, those problem areas, that they experience as they go along? So, that’s the bit I’m kind of interested in and where I think we can make a contribution.

Program team participant 6

Digital transformation may not necessarily be achieved easily. Apart from having a clear understanding and strategic planning for transformation, other aspects of society and organizations also need to go through change. For instance, digital transformation requires particular skills; staff either need to be trained or new employees need to be hired:

I think part of it is that a lot of frontline staff don’t even have the digital skills to pass on to the people they’re working with...I think a lot of charities don’t really have a grasp of the digital skill set of their staff. If you are a big social care charity with 2000 staff, we really need to get to grips with your status of digital skills and competence and where it’s lacking. How would you support them and how do you train them?

Program team participant 1

Digital transformation also requires cultural and structural changes in organizations:

I think there are those cultural issues and change management within services, and again, ensuring that the staff that are supporting people feel confident and equipped to use digital.

Program team participant 10

## Discussion

### Principal Findings

This study explored the benefits and challenges of the provision of digital technologies for digital inclusion, which aimed to support people at risk of DRDs through digital technology. Findings indicate that the provision of digital technologies offers many benefits to service users and providers. The technologies examined ranged from smartphones, tablets, and laptops to internet connectivity tools. We identified 3 main categories of perceived benefits for these devices: increased connectivity for informal support, increased access to services, and improved well-being.

This research found that provision of such devices and tools allowed service users to connect with others, overcome loneliness, create friendships, and build a sense of community, thus reducing the digital divide. Participants provided examples of connectivity to family and friends, as well as the importance of connectivity to a range of harm reduction and support services. Increased access to services was another benefit of digital devices, as described by both service users and providers, as people who were originally excluded from receiving information and support, due to a lack of access to technology, had an increased chance of being engaged in receiving such services. In most cases, they described better access to existing services, such as more readily available information or access to support workers, and better identification of existing events through web-based news, email, and media.

A novel finding was that digital technologies were seen to have significant potential in improving the well-being of the community by improving mental health and emotional well-being. Participants noted that access to digital technology provided a way to improve self-esteem and confidence. Some also noted that they used digital technologies provided by the program to learn new skills, which appeared to improve their mental well-being.

In general, the provision of digital technologies led to improved access to existing services and sources of support and information. At the time of this research, only in occasional cases, new services were designed, or existing services were somewhat transformed to web-based services. However, such cases had significant potential; for instance, as a participant highlighted because of new service provisions, 6 potential cases of prevention of overdose death were reported.

While the provision of technology has many potential benefits, we also identified technological, people-related, organizational, and macroenvironmental challenges that led to limited adoption and effective use of technology. Examples of the most evident challenges were inadequate digital literacy of service users (which could have other negative knock-on effects such as short intervals of engagement and periods of stress), technology usability issues, service providers being under pressure and being underresourced leading to limited planning for the offering of new services and effective adoption of technologies, and negative public and media perceptions of the program due to the particular context—drug use.

### Comparison With Prior Work

These findings contribute novel information in several ways. Our findings contribute to the literature on the potential benefits of digital technologies to tackle drug overdose by identifying 3 categories of benefits. Previous studies, such as McClure et al [30] and Ozga et al [31], found that there are still many concerns about the digital divide for marginalized populations, and our study shows that national programs that provide technologies can help mitigate some of these problems by providing better access to informal support, better access to existing formal services, and offering new or transformed services. This supports studies that show that digital technologies can be effective in treating drug use disorders [32,33]. While adoption of digital technologies is useful, our study found that implementation of technology alone is not sufficient for tackling the immense problems of drug overdose. Participants noted that many organizations were using some digital technology for providing activities and support to service users, but that was not transforming their services. The findings highlight the need for new value creation to enable new pathways such as new digital services. For instance, digital technologies can be used to redesign processes to streamline the existing ones or allow organizations to offer new services that are not offered if digital technologies were unavailable, such as remote consultations. Hence, digital transformation, a change process in organizations or societies driven by the use of digital technologies [34], is needed to better tackle such a complex issue. Digital transformation is not just about adopting digital technologies but also about redesigning and rethinking the existing processes to offer new and improved services [34]. Our evaluation confirmed that in drug harm reduction services, the digital transformation process requires particular skills, cultural and structural changes in organizations [29], and the transformation of society as a whole [35].

Digital transformation is the process of integrating digital technologies into an organization’s operations, strategies, and services to drive innovation, efficiency, and improved outcomes [19]. For drug service providers, digital transformation involves leveraging digital tools and technologies to enhance the delivery of addiction treatment and support services. We integrated a conceptual framework provided by Daneshvar et al [27] to explore the relationship between digital technology and the services provided to determine how far along the journey the sector is in relation to digital transformation. We show that currently, in most cases, existing digital tools are used to deliver existing services (in a digital form). Only very few new services were offered because of adopting digital tools (both existing and new digital tools). Hence, we propose that in the field of prevention of overdose death, we are only at the early stages of digital transformation. There are several reasons for this as either identified in previous research or by our study. For instance, many organizations do not have the required digital infrastructures [36], there are challenges with regard to data security and privacy [37-39], limited digital literacy [40], and technology usability in health and care [41,42]. Our study contributes to these areas of concern by indicating ways in which these challenges can be addressed. For example, training programs offered by service providers (eg, setting up accounts and email addresses to use social media) can be a very effective approach to boost the confidence of service users, which further improves their engagement with technology. We found different preferences and feelings expressed toward different devices offered by the digital inclusion program. Smartphones and tablets were considered more learnable and usable to those who were less digitally proficient, as opposed to laptops and Chromebooks. Thus, the usability of digital technologies was important. Hence, digital transformation programs need to consider these aspects when designing or transforming services.

### Implications for Policy, Practice, and Research

The findings underscore the necessity for policies and programs aimed at addressing digital inclusion to ameliorate the digital divide among marginalized populations. Provision of access to devices and internet connectivity can facilitate social connections and access to support services for people who use drugs. Government policies are essential to ensure the secure and ethical use of digital technologies in the health care sector, encompassing protections for sensitive health data. Regulatory frameworks surrounding privacy and consent should be fortified. Harm reduction organizations could integrate digital technologies, such as telehealth, mobile health apps, and web-based tools, into service delivery models where appropriate. Digital modalities can enhance convenience, accessibility, and engagement. Service providers could necessitate training in both using new technologies and imparting digital skills to service users. Supporting digital literacy and usability is imperative for successful adoption. Practitioners must consider user preferences and be cautious of a one-size-fits-all approach to technology.

There is a need for more rigorous studies and formative evaluations to establish the efficacy of specific digital health interventions for substance use disorders, particularly newer mobile health apps and wearable biosensors. Research should investigate how technology influences intended treatment outcomes. Qualitative data on user experiences are also invaluable. Cost-effectiveness studies would bolster decision-making regarding investment in digital modalities versus traditional delivery methods. Further exploration of barriers to digital transformation of addiction services could steer stakeholders toward transitioning from simple digitization of existing services to more innovative care models. Quantitative studies, such as randomized controlled trials, might be possible in the future to compare outcomes of those with particular digital technologies. In addition, a mixed methods study could investigate whether the availability of technology might lead to unintended consequences, such as facilitating connections with drug providers.

### Strengths and Limitations

This work is timely and coincides with the urgent impetus to respond to DRD and significant political appetite in Scotland, as well as the enhanced availability of digital health care funding because of COVID-19 [20,36]. To the knowledge of the authors, this is the first study to attempt to combine primary data regarding the digital inclusion for harm reduction and prevention of overdose death from the perspective of service users, service providers, and the program team. The specific strengths of this study lie in those areas that were identified as complementing academic literature. For example, the themes presented here were intentionally selected for their ability to elucidate the sociotechnical task faced by technologists and intervention implementation teams. The subtleties of the challenge, such as the delicate balances required to achieve acceptability, may be highly specific to this population. The opportunity presented to support the development of the TPOM for this sector and sociotechnical theory more broadly was also welcomed. Another strength of the work is the total number of interviews (n=47) conducted for this study, which provides a comprehensive perspective on the topic across stakeholders.

A limitation of the work is that our sample population consisted of people who participated in the program and therefore provided with access and support to digital tech. While we tried to speak to people who declined to participate in DLS, there was no engagement from them. Participation from individuals and groups involved in DRD prevention at the level of policy and elected officials would have added finer details concerning the macroenvironment and, thus, greater strategic insight into the overall feasibility of digital programs.

### Conclusions

Digital inclusion programs can offer a platform for engagement of individuals at risk of drug-related harm to offer them a space for accessing informal support, formal existing services, or, in some cases, experiencing and benefiting from new services. However, challenges remain, including service users' concerns regarding data security, digital literacy, and technology usability issues. Addressing these concerns necessitates a training program for both service providers and users to enable them to fully embrace the opportunities provided by digital connections, as well as initiating a cultural and societal shift toward the development and use of technologies to tackle DRDs. Moving beyond device provision, such programs should focus on the development of new digital services. Digital transformation is complex and may take a long time to achieve, but it can have a significant impact on preventing DRDs. In the modern landscape of health and social care, access to digital technology for all is no longer a luxury but an essential necessity.

## Appendix

Multimedia Appendix 1 Topic guide.

## Abbreviations

DLS: Digital Lifelines Scotland
DRD: drug-related death
TPOM: Technology, People, Organization, and Macroenvironment
