# Supplementary material for: From Digital Inclusion to Digital Transformation in the Prevention of Drug-Related Deaths in Scotland: Qualitative Study
Source: J Med Internet Res. 2024 Sep 24;26:e52345. doi: 10.2196/52345 (PMC11462095; doi:10.2196/52345)
Supplement: Multimedia Appendix 1 [file jmir_v26i1e52345_app1.docx]

**Interview Topic Guide - 1**

**Service Users/People Who Use Drugs**

COVER CONSENT, GROUND RULES, CONFIDENTIALITY, RECORDING, PUTTING PEOPLE AT EASE ETC.

1. Do you receive any digital services/devices from any service providers?
   1. If yes,
      1. What services/devices do you receive and how do you feel about it in terms of: how easy it is to use or learn to use as well as security and confidentiality of data, confidentiality, etc.
      2. When did you start receiving the services and did they change over time?
2. What benefits have you experienced from using digital technology so far?
3. Thinking about the devices mentioned above, what do you typically use these for e.g. communication, information access etc?
4. What worked well for you in terms of devices and/or digital services? What worked less well? Why?
5. Please tell us how you feel your digital skills/ confidence? Do you think these could be improved? How?
6. Have you been involved in getting digital services started or used more? At what stages and in what ways?
7. Do you think the changes to digital technology have changed the relationships between you and the service providers?
8. Is there anything else you’d like to add?

**Interview Topic Guide - 2**

**Service Providers**

COVER CONSENT, GROUND RULES, CONFIDENTIALITY, RECORDING, PUTTING PEOPLE AT EASE ETC.

1. Can you please tell me about how you deliver digital services/devices to any service users through the Digital Lifeline fund? What services/devices do you offer and how do you feel about it in terms of:

usability, learnability, security, accessibility, confidentiality, etc.

1. When did you start offering the services and did they change over time?
2. What are the different uses of digital technologies you are offering– e.g. communication, information access, etc.
3. What types of benefits have you identified from the use of digital technology so far? What about challenges?
4. Were there any unintended consequences in the use of digital services/provision of devices, either positive or negative?
5. What worked well in terms of digital technology? What worked less well? Why?
6. Please tell us how you feel about your digital skills and/or confidence? Do you think they could be improved? How?
7. Have you been involved in the implementation, adoption, and optimisation of digital services? At what stages and in what ways?
8. Has the digital initiative changed relationships between you and the service users?
9. In terms of the implementation of digital services:
   1. Are management structures adequate to support services offered?
   2. Is the training provided adequate, realistic and effective?
   3. Are resources provides (prompts: including technology, supporting change) adequate?
10. With regards to the Digital Lifelines Scotland programme, is there anything you would have wanted to be done differently? E.g. addition of new services etc.
11. Is there anything else you’d like to add?

**Interview Topic Guide - 3**

**Programme team and board**

COVER CONSENT, GROUND RULES, CONFIDENTIALITY, RECORDING, PUTTING PEOPLE AT EASE ETC.

1. From your point of view can you explain the different parts of the programme? What are the benefits and challenges?
2. In terms of programme delivery, what do you think worked well? What worked less well?
3. Were there any unintended consequences in the use of digital services, either positive or negative?
4. In terms of implementation of digital services:
   1. Are management structures adequate to support services offered?
   2. Is the training provided adequate, realistic, and effective?
   3. Are the resources provided (including technology, change management and maintenance) adequate?
5. How is the Digital Lifelines programme viewed by the media and by the public? How does the organisation view/manage media relations?
6. What benefits do policymakers expect from digital technology?
7. With regards to DLS programme, is there anything you would have wanted to be done differently?
8. Is there anything else you’d like to add?
